# Measuring Public Speaking Anxiety: Self-report, behavioral, and physiological

**DOI:** 10.1177/0145445521994308

**Published:** 2021-02-16

**Authors:** Ana Gallego, Louise McHugh, Markku Penttonen, Raimo Lappalainen

**Affiliations:** 1 University of Jyväskylä, Jyväskylä, Finland; 2 University College Dublin, Dublin, Ireland

**Keywords:** public speaking anxiety, social anxiety, distress tolerance, speech challenge, behavioral assessment task, physiological reactivity

## Abstract

Self-reports are typically used to assess public speaking anxiety. In this study, we examined whether self-report, observer report, and behavioral and physiological reactivity were associated with each other during a speech challenge task. A total of 95 university students completed a self-report measure of public speaking anxiety before and after the speech challenge. Speech duration (i.e., behavioral measure), physiological reactivity, as well as speech performance evaluated by the participants and observers were also recorded. The results suggest that self-reported public speaking anxiety predicts speech duration, as well as speech quality, as rated by the participants themselves and observers. However, the physiological measures were not associated with self-reported anxiety during the speech task. Additionally, we observed that socially anxious participants underrate their speech performance in comparison to their observers’ evaluations.

## Introduction

Speaking in public is the most commonly reported fear in the general population (Dwyer & Davidson, 2012; Sawyer, 2016). Public speaking anxiety is considered a social anxiety disorder and refers to the anxiety that an individual experiences when giving a speech or preparing to speak in front of others. In Finland, one in three students report that speaking in public is a severe problem for them (Kunttu et al., 2017). In the U.S., more than 61% of university students note a fear of speaking in public (Dwyer & Davidson, 2012). However, public speaking is an important skill for undergraduate students to learn and practice as they progress through their education and careers. To that end, speaking in public is a common requirement in undergraduate courses that encourages students to present their work and ideas to increase competency. For individuals who experience public speaking anxiety, speaking in public can have a negative impact on both their physical and emotional wellbeing. Public speaking anxiety symptoms can manifest in many different ways, such as bodily sensations, irrational thinking (e.g., “I’m concerned I’ll appear incompetent”), altered emotions, and avoidant behavior (Daly et al., 1997).

Self-report methods are the most commonly used measure in psychology (Paulhus & Vazire, 2009). This popularity is based on a number of advantages, including the method’s low cost and the opportunity to administer it in a mass testing session, where hundreds of variables can easily be collected at once. However, although some studies suggest that self-reports are adequate indexes of actual behaviors and attitudes (e.g., Corral-Verdugo & Figueredo, 1999), other studies suggest the opposite (e.g., Fuj et al., 1985). In the public speaking anxiety literature, self-reports are the most widely used tool to assess speech anxiety. Still, speech challenges (i.e., behavioral assessment task, BAT) are frequently used to assess avoidant behavior/distress tolerance in public speaking (Beidel et al., 1989). Physiological measures have also commonly been used to assess physiological reactivity while giving a public speech (Sawyer & Behnke, 1999). Subsequently, previous studies have explored the interrelationships among public speaking anxiety components to evaluate the validity of using different systems to assess public speaking anxiety (Bodie, 2010). In contrast, in a review of the public speaking anxiety literature, Clevenger (1959) suggests that even when different measures (e.g., cognitive, physiological, and behavioral) report high reliability, these measures are not meaningfully correlated. After approximately 30 years of research, McCroskey (1984) states that self-reports, physiological arousal indicants, and observer ratings of public speaking anxiety do not measure the same thing. In sum, since Clevenger’s (1959) statement, the concern about whether these systems are related has been a major concern in the public speaking anxiety literature (Bodie, 2010). Yet, the interrelationship among the different measures that assess speech anxiety is not fully understood even in the present day. Furthermore, research has demonstrated that the effectiveness of psychological interventions in the reduction of social and public speaking anxiety differs depending on the measurements used to assess it (Allen, 1989; Ebrahimi et al., 2019). For instance, several studies have found that the effectiveness of interventions evaluated through self-reports is greater compared to that of physiological and behavioral measures (Heimberg et al., 1990). Therefore, this current gap in the literature could mislead both researchers and practitioners to misidentify levels of public speaking anxiety, resulting in erroneous conclusions and interpretations.

Given the fact that different measures (self-report, behavioral, and physiological) might capture different facets or skills during a speech challenge, it is important to understand how these different measures are related to each other and speech performance. Thus, we investigated whether four components of public speaking were related to each other during a speech challenge task. These components were self-report, observer report, and behavioral and physiological reactivity. Based on the previous literature, we predicted that self-reported public speaking anxiety is unrelated to physiological measures. To the best of our knowledge, there is no previous research about the connection between speech duration and physiological measures. In addition, we predicted that the speech performances evaluated by the participants and external observers are connected to each other, but that there is a significant difference in the level of evaluation between them. We expected this result since previous studies indicate that participants with social anxiety underestimate their speech performance in comparison to external observers (Rapee & Lim, 1992).

## Method

### Participants

The participants (*n* = 106) were university students recruited from the Department of Education and the Language Centre at the University of Jyväskylä. These students were recruited from introductory courses that aimed to improve communication skills. At the start of each course, the students received the following information: “The study is related to public speaking and communication skills. You will have the possibility to give a speech in front of a camera; meanwhile, your physiological reactivity will be recorded. In addition, you will fill in some psychological questionnaires. For this, you will not need to prepare anything beforehand.” After this, the courses’ principal teacher sent the students an online scheduling tool through which they could voluntarily sign up for the experiment. All participants were undergraduate students. For ethical reasons, we conducted the experiment with all the students that signed up. However, we excluded from the analyses participants who were taking psychogenic medication or did not fill in their personal information (*n* = 11). This resulted in a final sample of 95 participants (53% female). Their ages ranged from 20 to 46 (*M* = 24.61, *SD* = 4.77), and the amount of years they had been studying at the university ranged from 1 to 8 (*M* = 2.61, *SD* = 1.42).

### Procedure

The experiment was conducted individually at the Department of Psychology. In the experiment room, an individual participant sat in a chair in front of a video camera situated at eye level. Behind the camera was a 65-inch TV screen, and behind the participant there was an amplifier (BrainVision QuickAm with 32 EEG and 8 physiological channels) to record electrodermal and electrocardiogram activity. Next to the participant were the self-report questionnaires and a pen. The researcher was in an adjacent room equipped with two computers and a laptop. One of the computers was used to play the audio-recorded instructions, the other computer managed the BrainVision recorder program, and the laptop was used to play a video-recorded audience on the TV screen in front of the participant. To monitor the participants and communicate with them, there was a 23” TV screen and microphone connected to a speaker in the participant room.

**Figure 1.**
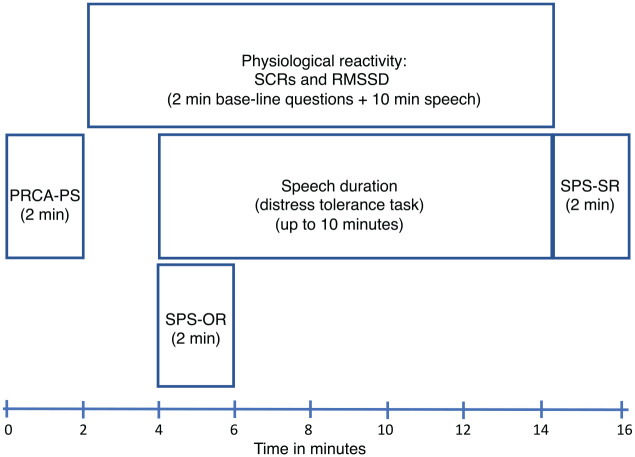
Procedure timeline. *Note*. PRCA-PS = self-reported public speaking; SCRs = skin conductance responses; RMSSD = heart-rate variability-root mean square of successive RR interval differences; SPS-SR = self-perceived speech performance; SPS-OR = others-perceived speech performance.

The experiment consisted of six phases. First, the participants were asked to fill in their informed consent and background/personal information. Second, they completed the self-report questionnaires (for more information, see the Measures section). In addition, the recording of physiological activity (heart rate and electrodermal activity) started at this phase and continued during the following phases. Third, the participants were asked neutral questions to use as a baseline for their physiological measurements. Fourth, as a behavioral task, the participants were instructed to give an impromptu 10-minute speech about themselves, including their strengths and weaknesses, in front of the camera and video-recorded audience. Fifth, before beginning to talk, the participants were allowed 3 minutes to plan their speech. Sixth, the participants gave their speeches (Figure 1). The termination of the speech task before the end of the 10-minute period was assessed as avoidance behavior and the total amount of time (speech duration) that they spoke as distress tolerance (England et al., 2012; Gallego et al., 2020).

### Measures

#### Self-report measures

##### Personal Report of Communication Apprehension, Public Speaking Subscale (PRCA-PS)

This subscale includes six items (e.g., “My thoughts become confused and jumbled when I am giving a speech”). Each item is graded on a 5-point Likert scale from 1 (*strongly agree*) to 5 (*strongly disagree*). Lower scores indicate less apprehension about speaking in public. Scores can range from 6 to 30. Moderate levels of anxiety toward speaking in public range from 13.75 to 20.75, and high levels oscillate from 20.75 to 30. The validity and reliability of this scale are well known. In a previous study, the subscale’s Cronbach’s alpha shows an excellent reliability for all items (McCroskey et al., 1985). In the present study, the PRCA-PS demonstrated good internal consistency (Cronbach’s alpha = .85; McCroskey, 1982).

##### Social Performance Scale Self-Reported Version (SPS-SR)

After the speech challenge, the participants assessed their perceived speech performance through the self-reported version of the SPS. This scale includes 17 items rated on a 5-point scale from 0 (*not at all*) to 4 (*very much*). Final scores range from 0 to 68, with greater scores indicating a higher-quality perceived performance. The scale’s validity and reliability have been proven in previous research (Rapee & Lim, 1992; Tutino et al., 2020). In this study, the internal consistency was .88 (Cronbach’s alpha; Rapee & Lim, 1992) .

##### Visual Analog Scales (VAS)

In this study, the students answered the following question: “How *uncomfortable* do you feel to give the speech?” The participants were instructed to indicate how they felt by placing an X on a printed line that ranged from 0 (*not uncomfortable at all*) to 10 (*extremely uncomfortable*). According to Boonstra et al. (2014), a score ≤3.8 indicates mild symptoms, between 3.9 and 5.7 moderate, and scores ≥5.8 severe.

#### Observers’ evaluation

##### Social Performance Scale Other-Reported Version (SPS-OR)

After the experiment, independent raters evaluated the video-recorded speeches. The SPS-OR was used to assess speech performance as perceived by these external evaluators. The scale consists of 17 items that gauge performance features (e.g., voice clarity, fidgeting). Each item is rated on a 5-point Likert scale. Scores can range from 0 to 68, with higher numbers indicating a better performance. Research has shown that the SPS-OR’s rating is valid and reliable (Rapee & Lim, 1992; Tutino et al., 2020).

Two independent evaluators rated each video speech. During the training phase, an expert from the Language Centre of the University of Jyväskylä trained the observers to assess the speakers’ performances. The expert and observers examined the SPS-OR together to have a common consensus on the items’ meaning. The expert and observers also evaluated a video sample together to reach agreement on the evaluation criteria. After that, the observers evaluated another video sample to check the ratings’ consistency. The videos used during the training phase were selected from the piloting period of this study and were not included in this study’s analyses (i.e., the videos were only used for training purposes). The videos included in the results of this study lasted a maximum of 10 minutes. However, due to limited resources, all videos were edited to 2 minutes. We selected the first 2 minutes of each speech for two reasons. First, there was a large variation in how long the participants gave their speeches, and all the participants talked for at least 2 minutes. Therefore, that was the period with the most reliable data. Second, research has identified four characteristics or phases during public speaking events: (1) anticipation—pre-speech, (2) confrontation—the first speaking minute, (3) adaptation—the last speaking minute, and (4) release—time between the end of the speech and 1 minute post-speech (Behnke & Carlile, 1971; Carlile et al., 1977). Both of these reasons resulted in our decision to only include the confrontation phase. After the training phase was successfully completed, the rating phase took place. The video ratings were conducted in eight rounds, and the observers reviewed the reliability of the ratings on a rounds basis. In the first 7 rounds, each reviewer rated 10 videos, 6 of which were the same to calculate reliability. In between rounds, there was a practice evaluation to help maintain reliability. During these practice evaluations, the observers independently examined the same samples and then discussed their interpretations together. The evaluations done in the practice evaluation phase were not included in the results. The Cronbach’s alpha for the two observers was 0.96.

#### Behavioral measures

##### Speech challenge

The participants were requested to give an impromptu speech: “I would like to invite you to give a 10-minute speech about yourself, your strengths, and weaknesses. I hope that you can speak for as long as possible. I will let you know when the time is up. If you decide to end your speech earlier, please say out loud, ‘I want to stop.’ Try to continue the speech if you can, even if you’re not sure what you would say next. You can stop if necessary if you are anxious and you cannot continue. Now you have 3 minutes to think about what you want to say in your speech. If you want, you can write down what you want to say.” The length of the speech provided a behavioral measure of avoidance/distress tolerance. The maximum duration for the speech was 10 minutes. Prior research proposes that ending a speech prematurely can be interpreted as an attempt to escape the anxiety that arises when speaking in front of others (England et al., 2012; Gallego et al., 2020). Accordingly, speech duration represented a behavioral measure of distress tolerance.

#### Physiological measures

*Electrodermal activity* (EDA) was measured with two skin-conductance electrodes (Ag/AgCl, EL 507, BioPac Systems) positioned on the participants’ non-dominant palm, one placed beneath the thumb and the other under the fourth and fifth digits. The participants were asked to hold that hand on the chair’s armrest without moving it. Skin conductivity was registered using a galvanic skin response module (Brain Products) that determined conductivity by directing a 0.5 V voltage between the electrodes and measuring the conductivity changes with a direct current (DC) amplifier. Skin conductance was recorded in DC mode using a BrainVision QuickAmp. The signal was low-pass filtered at 400 Hz and sampled at 1,000 Hz using the BrainVision Recorder 1.20.0801 program.

*Electrocardiograms* were registered using three electrodes (Ag/AgCl, Ambu Neuroline 710). One of the electrodes was situated on the left shoulder, another electrode was placed beneath the clavicle on the right side, and the last electrode was placed on the left side above the bottom ribs, forming a triangle encompassing the heart. The signal was high-pass filtered at 0.5 Hz, low-pass filtered at 400 Hz, and sampled at 1,000 Hz using the QuickAmp and Recorder program.

#### Data analysis plan

EDA was analyzed with MATLAB R2014a using Ledalab V3.4.9 (Benedek & Kaernbach, 2010). In this regard, rapid changes in EDA (skin conductance responses, SCRs) were separated from slowly varying activity (skin conductance level, SCL). Subsequently, the mean SCR values were computed for every phase of interest, depicting sympathetic nervous system activation. Heart-rate variability (HRV) was assessed from an electrocardiogram with Kubios HRV Premium programs (www.kubios.com). At first, the programs expunged automatically possible artifacts and counted successive interbeat intervals (RR intervals). The HRV index used in this study was the square root of the mean squared differences between successive RR intervals (RMSSD). The HRV index was computed for each phase of interest.

For the statistical analyses, both RMSSD and SCRs were normalized with a 2-minute baseline phase. In this phase, the participants were asked basic questions (e.g., “What is your name?”; “Where were you born?”; “Where are you from?”; and “What is your favorite season of the year?”). Changes in physiology during the speech were calculated by computing relative changes from the baseline using the following formula: (speech–speech baseline)/speech baseline (as a percentage). The analyses were conducted with these normalized variables to give consideration to the individual variation in physiological reactivity. All statistical analyses were performed using IBM SPSS Statistics 24. The correlations between the variables were investigated using the Pearson correlation test. A small correlation ranged from *r* = 0.10 to 0.30, a moderate correlation from *r* = 0.31 to 0.50, and a high correlation from *r* = 0.51 to 1 (Cohen, 1992).

## Results

**Figure 2.**
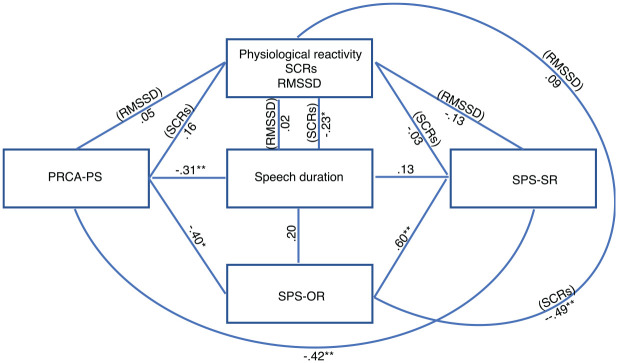
Correlations. *Note*. PRCA-PS = self-reported public speaking anxiety; SCRs = skin conductance responses; RMSSD = heart-beating-square root of the mean squared differences between RR intervals; SPS-OR = observer-evaluation of speech performance; SPS-SR = self-evaluation of speech performance; speech duration = public speaking distress tolerance.

In relation to how anxious the participants felt giving the impromptu speech, 57% reported high levels of anxiety, 20% moderate levels, and 22% lower levels (VAS). Regarding level of public speaking anxiety, in this study, 50% of the participants recorded having high anxiety, 42% moderate anxiety, and only 9% low anxiety. In the present study, the maximum speech length was 10 minutes, and the mean time that the participants used for the speech was 7.45 minutes (*SD* = 2.53; Table 1). The results of our study show that higher levels of self-reported public speaking anxiety (PRCA-PS) correlate with shorter speech duration (i.e., behavioral task of public speaking distress tolerance). This correlation is moderate (*r* = −.31, *p* < .01, *n* = 95). According to the results, there is no correlation between self-reported public speaking anxiety (PRCA-PS) and SCRs (*r* = .16, *n* = 92) or HRV (RMSSD; *r* = .05, *n* = 93). However, higher levels of self-reported public speaking anxiety moderately correlate with poor self-perceived speech performance (SPS-SR; *r* = −.42, *p* < .01, *n* = 95). Higher levels of self-reported public speaking anxiety also moderately correlate with poorer speech performance as perceived by external observers (SPS-OR; *r* = −.40, *p* < .05, *n* = 95). These results are summarized in Table 2 and Figure 2. In addition, the results show that there is a positive correlation between self- and others-perceived speech performance (*r* = .60, *p* < .01, *n* = 95), indicating that the better quality in speech performance evaluated by oneself, the better others might evaluate it. Nevertheless, a t-test identified a significant difference between self-perceived speech performance and speech performance as rated by external observers (*p* < .01), favoring the latter (self-performance *M* = 38.58, *SD* = 9.55, *N* = 103; others-performance *M* = 56.28, *SD* = 7.30, *N* = 43; Table 3). In relation to public speaking distress tolerance (i.e., speech duration), the data depicts that higher levels of distress tolerance correlate with lower levels of skin conductance activation (*r* = −23, *p* < .005, *n* = 95). Yet, there is no correlation with HRV (*r* = 0.02, *n* = 95). Furthermore, there is no correlation between speech duration and speech performance as evaluated by the participant, nor with speech performance as evaluated by observers.

**Table 1.** Descriptive Statistics.

	Minimum	Maximum	Mean (*SD*)	95% Confidence interval	
				Lower	Upper
PRCA-PS	11	30	20.5 (4.75)	19.56	21.54
RMSSD	−0.44	5.70	0.09 (0.64)	−0.01	0.24
SCRs	−0.77	2.91	−0.18 (0.48)	−0.27	−0.07
VAS1	0	10	5.76 (2.64)	5.21	6.28
SPS-SR	14	53	39.24 (9.52)	37.16	40.96
SPS-OR	37	67	56.42 (7.5)	54.13	58.63
Speech duration	1:12	10:00	7:45 (2:53)	7:06	8:20

*Note.* PRCA-PS = public speaking anxiety; RMSSD = heart-rate variability-root mean square of successive RR interval differences; SCRs = skin conductance responses; VAS1 = how uncomfortable does it make you feel to give the speech?; SPS-SR = self-perceived speech performance; SPS-OR = others-perceived speech performance.

**Table 2.** Correlations.

	RMSSD	SCRs	Speech duration	SPS-SR	SPS-OR	VAS1
PRCA-PS	.06	.16	−.31**	−.42**	−.40*	.48**
RMSSD	1	.14	.01	−.13	.10	−.05
SCRs		1	−.23*	−.03	−.49**	−.03
Speech duration			1	.13	.20	−.34**
SPS-SR				1	.60**	−.55**
SPS-OR					1	−.38*
VAS1						1

*Note*. PRCA-PS = public speaking anxiety; RMSSD = heart-rate variability-root mean square of successive RR interval differences; SCRs = skin conductance; SPS-SR = self-perceived speech performance; SPS-OR = others-perceived speech performance; VAS1 = how uncomfortable does it make you feel to give the speech?

* The correlation is significant at the 0.05 level. **The correlation is significant at the 0.01 level.

**Table 3.** Mean Score of Self- and Observers Ratings on Global and Specific Items on Public Speaking Performance.

Rating	SPS-OR		SPS-SR	
	*M*	*SD*	*M*	*SD*
Specific items	42.95	3.44	31.36	6.21
Global items	13.48	4.01	7.25	4.21
Total score	56.29	7.30	38.58	9.56

*Note*. SPS-SR = self-perceived speech performance; SPS-OR = others-perceived speech performance.

## Discussion

The present study aimed to examine the relationship between self-reported public speaking anxiety, a behavioral assessment of public speaking distress tolerance (i.e., speech duration), physiological reactivity during a speech challenge, and the quality of the speech as evaluated by both the participants and observers. The results depicted a negative and moderate correlation between speech duration and self-reported public speaking anxiety, suggesting that students who report high levels of public speaking anxiety also give shorter presentations. This may be indicative of an avoidance strategy. In line with this postulate, previous studies empirically demonstrate that individuals with higher levels of experiential avoidance have lower distress tolerance (Feldner et al., 2006; Zettle et al., 2005). Thus, our study indicates that self-reported public speaking anxiety can predict actual avoidance behavior.

The present results also showed no correlation between self-reported public speaking anxiety and physiological arousal as measured during the speech challenge. Thus, the current data indicates that physiological reactivity during presentations is unrelated to experiences or self-reported level of public speaking anxiety. These observations are in line with Schachter and Singer (1962). They argue that high physiological arousal creates urges to understand and label the activity of the sympathetic nervous system. The label that an individual chooses depends on situational cues “as interpreted by previous experiences” (Schachter & Singer, 1962). Therefore, the researchers suggested hat an emotion is not fully explained by physiological arousal or cognitive perception alone, but the coaction of both. According to Behnke and Beatty (1981), public speaking anxiety can be understood, in part, as the predisposition to label the physiological arousal that arises when speaking in front of others as anxiety. Yet, for speakers for whom anxiety is not an appropriate label, they might understand physiological arousal as “exhilaration” or “facilitative energy,” the consequence of which being that they might not report high levels of public speaking anxiety. More recently, in the theory of constructed emotions, Barrett (2006) postulates that purely physical sensations in the body do not have objective meaning. For instance, a change in heart rate is not objectively or necessarily an emotion. As a result, the effectiveness of using solely physiological reactivity measures to detect indexes of public speaking anxiety is called into question. Our study, as well as others, have been unable to identify clear unique physiological correlates to self-reported public speaking anxiety. However, if physiological measures are used in conjunction with self-reported measures of the speech anxiety trait, they might account for a high proportion of the total variance of anxious arousal (i.e., panic during a speech; Finn et al., 2009). Furthermore, meta-analyses by Allen (1989) and Ebrahimi et al. (2019) indicate that research on the effectiveness of physiological measures has demonstrated a small effect on public speaking anxiety treatment and favors the use of self-reported measures. Still, other studies have detected treatment effects in the form of reduced levels of physiological reactions, even when reductions in self-reported levels do not occur (Kircanski et al., 2012; Niles et al., 2015). Further research is needed to clarify these mixed findings.

Moreover, the distress tolerance task (speech duration) correlated with skin conductance but not heart rate. According to Barry and Sokolov (1993), arousal is more closely expressed through increases in SCL (sweating) than cardiac acceleration. This could provide an explanation of why skin conductance in the current study related to the behavioral measure of public speaking distress tolerance but not to self-reported public speaking anxiety. Additionally, it is important to note that both speech duration and SCL are objective measures independent from the participants’ subjective experiences. Furthermore, the results of this study indicated that high levels of self-reported public speaking anxiety are associated with low-quality speech performance as evaluated by both the participants themselves and external observers. In line with this finding, previous studies have demonstrated that visualization techniques (i.e., imagining giving a speech) are effective in enhancing performance, as well as reducing public speaking anxiety (Ayres & Hopf, 1992). Therefore, it can be hypothesized that using techniques meant to enhance speech performance might reduce self-perceived speech anxiety as a collateral effect.

Additionally, the results revealed a highly significant correlation between the quality of the speech performance as rated by the participants themselves and the external observers, indicating that speeches evaluated as better by the observers were also evaluated as better by the speakers themselves and vice versa. The study Daly et al. (1989) reports similar results. However, our results also showed a significant difference between the participants’ and observers’ speech performance perceptions. This indicates that even when the speaker and external observers evaluated the speech performance as high, there was still a significant discrepancy between how skillful the speaker thought the speech was in comparison to the external evaluators. This indicates that the speakers underrated their speech performances in comparison to how the external observers evaluated their speeches. In line with this finding, Rapee and Lim (1992) report that socially anxious individuals show a greater discrepancy than normal controls between their speaking performance self-reports and observers’ ratings. This discrepancy between the speaker’s rating and that of the observers is larger for high-trait anxious speakers, as they rate their own performance more harshly then trained observers (Rapee & Lim, 1992).

There were a number of limitations to the current study. First, its design is correlational, and the results are thus based on the relationships between variables. Further research is needed to identify the exact causal nature of these relationships. Another limitation comes from the generalization of these results to a broader population. The current study was conducted with university students; as such, these findings are not directly transferable to clinical groups. Even so, this segment of the population was selected in view of the high rates of public speaking anxiety among undergraduate university students. Furthermore, in relation to the scales, only one questionnaire was used to assess self-reported public speaking anxiety. Still, the PRCA-PS is a well-documented and broadly used scale that has shown good psychometric properties. Additionally, physiological activity was only measured via HRV and EDA, which limits our conclusions on physiological reactivity. Future research could implement additional physiological measures, such as muscle activity, respiration, or neuroendocrine responses (i.e., cortisol levels). Moreover, although the current sample included a portion of students with severe/extreme levels of anxiety to give the requested impromptu speech, it is possible that many extremely anxious students did not volunteer for this study due to the nature of the topic. Consequently, the results could differ if a larger proportion of extremely anxious students is included. Accordingly, further studies are needed to clarify this issue.

In summary, the present study has a number of implications. First, according to our results as well as previous findings in the literature, it is not advisable to rely solely on physiological reactivity measures to assess public speaking anxiety. Arousal is not necessarily the same as anxiety (McCroskey, 1984). Therefore, physiological measures do not have sufficient face validity as indicators of public speaking anxiety to merit attention from researchers and practitioners concerned with this construct. On the other hand, many self-report measures in the public speaking anxiety literature have demonstrated both good reliability and validity. As stated by McCroskey (1984), self-report measurements with good psychometric properties, when utilized for legitimate purposes, can be invaluable to practitioners and researchers assessing public speaking anxiety. Using self-report measurements with poor psychometric properties, or such measures when other instruments could be more suitable, is therefore bad praxis that practitioners and researchers should avoid (McCroskey, 1984). Second, skin conductance reactivity is related to distress tolerance/avoidance. Thus, it can be hypothesized that increasing levels of distress tolerance and decreasing avoidance result in less physiological reactivity in anxiety-provoking situations (and vice versa). Third, since lower levels of self-reported public speaking anxiety are related to better-quality speech performance, it could be expected that decreasing levels of self-reported public speaking anxiety might result in increased speech performance quality. To conclude, our data proposes that self-reported public speaking anxiety predicts both avoidance behavior (speech duration) and speech performance, but it does not predict physiological reactivity while presenting.

## References

[bibr1-0145445521994308] Allen M. (1989). A comparison of self-report, observer, and physiological assessments of public speaking anxiety reduction techniques using meta-analysis. Communication Studies, 40(2), 127–139. 10.1080/10510978909368262

[bibr2-0145445521994308] Ayres J. Hopf T. (1992). Visualization: Reducing speech anxiety and enhancing performance. Communication Reports, 5(1), 1–10. 10.1080/08934219209367538

[bibr3-0145445521994308] Barrett L. F. (2006). Solving the emotion paradox: Categorization and the experience of emotion. Personality and Social Psychology Review, 10(1), 20–46. 10.1207/s15327957pspr1001_2 16430327

[bibr4-0145445521994308] Barry R. J. Sokolov E. N. (1993). Habituation of phasic and tonic components of the orienting reflex. International Journal of Psychophysiology, 15(1), 39–42. 10.1016/0167-8760(93)90093-5 8407432

[bibr5-0145445521994308] Behnke R. R. Beatty M. J. (1981). A cognitive-physiological model of speech anxiety. Communications Monographs, 48(2), 158–163. 10.1080/03637758109376055

[bibr6-0145445521994308] Behnke R. R. Carlile L. W. (1971). Heart rate as an index of speech anxiety. Speech Monographs, 38, 65–69. 10.1080/03637757109375689

[bibr7-0145445521994308] Beidel D. C. Turner S. M. Jacob R. G. Cooley M. R. (1989). Assessment of social phobia: Reliability of an impromptu speech task. Journal of Anxiety Disorders, 3, 149–158. 10.1016/0887-6185(89)90009-1

[bibr8-0145445521994308] Benedek M. Kaernbach C. (2010). A continuous measure of phasic electrodermal activity. Journal of Neuroscience Methods, 190(1), 80–91. 10.1016/j.jneumeth.2010.04.02 20451556 PMC2892750

[bibr9-0145445521994308] Bodie G. D. (2010). A racing heart, rattling knees, and ruminative thoughts: Defining, explaining, and treating public speaking anxiety. Communication Education, 59(1), 70–105. 10.1080/03634520903443849

[bibr10-0145445521994308] Boonstra A. M. Preuper H. R. S. Balk G. A. Stewart R. E. (2014). Cut-off points for mild, moderate, and severe pain on the visual analogue scale for pain in patients with chronic musculoskeletal pain. Pain, 155(12), 2545–2550. 10.1016/j.pain.2014.09.014 25239073

[bibr11-0145445521994308] Carlile L. W. Behnke R. R. Kitchens J. T. (1977). A psychological pattern of anxiety in public speaking. Communication Quarterly, 25(4), 44–46. 10.1080/01463377709369272

[bibr12-0145445521994308] Clevenger T. (1959). A synthesis of experimental research in stage fright. Quarterly Journal of Speech, 45, 134–145. 10.1080/00335635909385732

[bibr13-0145445521994308] Cohen J. (1992). Statistical power analysis. Current Directions in Psychological Science, 1(3), 98–101. 10.1111/1467-8721.ep10768783

[bibr14-0145445521994308] Corral-Verdugo V. Figueredo A. J. (1999). Convergent and divergent validity of three measures of conservation behavior: The multitrait-multimethod approach. Environment and Behavior, 31(6), 805–820. 10.1177/00139169921972353

[bibr15-0145445521994308] Daly J. A. McCroskey J. C. Ayres J. Hopf T. Ayres D. M. (1997). Avoiding communication: Shyness, reticence, & communication apprehension (2nd ed.). Hampton Press.

[bibr16-0145445521994308] Daly J. A. Vangelisti A. L. Lawrence S. G. (1989). Self-focused attention and public speaking anxiety. Personality & Individual Differences, 10, 903–913. 10.1016/0191-8869(89)90025-1

[bibr17-0145445521994308] Dwyer K. K. Davidson M. M. (2012). Is public speaking really more feared than death? Communication Research Reports, 29(2), 99–107. 10.1080/08824096.2012.667772

[bibr18-0145445521994308] Ebrahimi O. V. Pallesen S. Kenter R. M. F. Nordgreen T. (2019). Psychological interventions for the fear of public speaking: A meta-analysis. Frontiers in Psychology, 10, 488. 10.3389/fpsyg.2019.00488 30930813 PMC6428748

[bibr19-0145445521994308] England E. L. Herbert J. D. Forman E. M. Rabin S. J. Juarascio A. Goldstein S. P. (2012). Acceptance-based exposure therapy for public speaking anxiety. Journal of Contextual Behavioral Science, 1(1–2), 66–72. 10.1016/j.jcbs.2012.07.001

[bibr20-0145445521994308] Feldner M. T. Hekmat H. Zvolensky M. J. Vowles K. E. Secrist Z. Leen-Feldner E. W. (2006). The role of experiential avoidance in acute pain tolerance: A laboratory test. Journal of Behavior Therapy and Experimental Psychiatry, 37(2), 146–158. 10.1016/j.jbtep.2005.03.002 15882839

[bibr21-0145445521994308] Finn A. N. Sawyer C. R. Schrodt P. (2009). Examining the effect of exposure therapy on public speaking state anxiety. Communication Education, 58(1), 92–109. 10.1080/03634520802450549

[bibr22-0145445521994308] Fuj E. T. Hennessy M. Mak J. (1985). An evaluation of the validity and reliability of survey response data on household electricity conservation. Evaluation Review, 9(1), 93–104. 10.1177/0193841x8500900106

[bibr23-0145445521994308] Gallego A. McHugh L. Villatte M. Lappalainen R. (2020). Examining the relationship between public speaking anxiety, distress tolerance and psychological flexibility. Journal of Contextual Behavioral Science, 16, 128–133. 10.1016/j.jcbs.2020.04.003

[bibr24-0145445521994308] Heimberg R. G. Dodge C. S. Hope D. A. Kennedy C. R. Zollo L. J. Becker R. E. (1990). Cognitive behavioral group treatment for social phobia: Comparison with a credible placebo control. Cognitive Therapy and Research, 14(1), 1–23.

[bibr25-0145445521994308] Kircanski K. Lieberman M. D. Craske M. G. (2012). Feelings into words: Contributions of language to exposure therapy. Psychological Science, 23(10), 1086–1091. 10.1177/0956797612443830 22902568 PMC4721564

[bibr26-0145445521994308] Kunttu K. Pessonen T. Saari J. (2017). Ylioppilaiden Terveydenhoitosäätiön Tutkimuksia 48.

[bibr27-0145445521994308] McCroskey J. C. (1982). An introduction to rhetorical communication (4th ed.). Prentice-Hall.

[bibr28-0145445521994308] McCroskey J. C. (1984). Self-report measurement. In Daly J. A. McCroskey J. C. (Eds.), Avoiding communication: Shyness, reticence, and communication apprehension (pp. 81–94). Sage.

[bibr29-0145445521994308] McCroskey J. C. Beatty M. J. Kearney P. Plax T. G. (1985). The content validity of the PRCA-24 as a measure of communication apprehension across communication contexts. Communication Quarterly, 33(3), 165–173. 10.1080/01463378509369595

[bibr30-0145445521994308] Niles A. N. Craske M. G. Lieberman M. D. Hur C. (2015). Affect labeling enhances exposure effectiveness for public speaking anxiety. Behaviour Research and Therapy, 68, 27–36. 10.1016/j.brat.2015.03.004 25795524

[bibr31-0145445521994308] Paulhus D. L. Vazire S. (2009). The self-report method. In Robins R. W. Farley R. C. Krueger R. F. (Eds.), Handbook of research methods in personality psychology (pp. 224–239). The Guilford Press.

[bibr32-0145445521994308] Rapee R. M. Lim L. (1992). Discrepancy between self- and observer ratings of performance in social phobics. Journal of Abnormal Psychology, 101(4), 728–731. 10.1037/0021-843x.101.4.728 1430614

[bibr33-0145445521994308] Sawyer C. R. (2016). Communication apprehension and public speaking instruction. In Witt P. (Ed.), Communication and learning (pp. 397–426). De Gruyter.

[bibr34-0145445521994308] Sawyer C. R. Behnke R. R. (1999). State anxiety patterns for public speaking and the behavior inhibition system. International Journal of Phytoremediation, 21(1), 33–41. 10.1080/08934219909367706

[bibr35-0145445521994308] Schachter S. Singer J. (1962). Cognitive, social, and physiological determinants of emotional state. Psychological Review, 69(5), 379–399. 10.1037/h0046234 14497895

[bibr36-0145445521994308] Tutino J. S. Ouimet A. J. Ferguson R. J. (2020). Exploring the impact of safety behaviour use on cognitive, psychophysiological, emotional and behavioural responses during a speech task. Behavioural and Cognitive Psychotherapy, 48, 557–571. 10.1017/S135246582000017X 32301412

[bibr37-0145445521994308] Zettle R. D. Hocker T. R. Mick K. A. Scofield B. E. Petersen C. L. Song H. Sudarijanto R. P. (2005). Differential strategies in coping with pain as a function of level of experiential avoidance. The Psychological Record, 55(4), 511–524. 10.1007/bf03395524

